# A taxonomy of athletic values and implications for clinical practice

**DOI:** 10.3389/fpsyg.2025.1462295

**Published:** 2025-02-24

**Authors:** Jordan Collins, Zachary C. Merz, Jeffrey D. Gfeller, Michael Ross

**Affiliations:** ^1^Department of Psychology, Saint Louis University, St. Louis, MO, United States; ^2^Moses H Cone Memorial Hospital, Greensboro, NC, United States

**Keywords:** sport psychology, mindfulness acceptance and commitment, value-driven behavior, performance enhancement, athletic values

## Abstract

**Introduction:**

The current study surveyed collegiate student-athletes regarding their perceived level of importance surrounding 30 previously derived and empirically obtained athletic values to improve viability of sport psychological practices.

**Methods:**

A total of 162 student-athletes enrolled in a private Midwestern NCAA Division 1 university within the United States of America completed tasks asking them to sort and rate utilized values based upon perceived importance surrounding athletic performance and sustained excellence.

**Results:**

Results revealed a hierarchy of athletic values, favoring intrinsic values, useable when emphasizing the importance of value-driven behavior in applied sport psychological practices. Minimal differences were seen across gender, ethnicity, sport classification, and other comparative groups.

**Discussion:**

Current results may help inform sport psychological practice while working within value-based frameworks.

## Introduction

Values represent personal beliefs that can directly influence inward thoughts and outward behaviors (Rokeach, 1973). While the concept of values is seemingly universal, they are perceived differently by each individual, group, and culture. Thus, values may have different meaning to different persons. Understanding personal values and fostering appropriate value-driven behavior is a common therapeutic technique throughout the field of clinical psychology [see Acceptance and Commitment Therapy (ACT)] (Hayes and Strosahl, 2004). It is also a vital aspect of clinical sport psychological practice, holding a prominent place within the Mindfulness, Acceptance, and Commitment (MAC) (Gardner and Moore, 2004) sport psychological intervention approach. Despite this, to the authors’ knowledge, there have been no attempts to delineate a taxonomy of values used by athletes for sport performance enhancement. The current study sought to identify and examine such athletic values.

### Introduction to values

Values can be described as trans-situational motivations which serve as guiding principles for individuals’ lives. From an early age, children learn values not only in the home, but from outside influences as well. For example, Cranmer and Myers (2017) found that the behaviors, attitudes, and values of parents are regularly internalized, adopted, and replicated by their children. Halstead and Taylor (2000) also found that children are exposed to several external factors, cultivated from the interactions with mass media and other agencies and situations that influence personal values. These values are said to change over the lifespan and be further influenced by historical events, life stages, and emotional development, with much of this change occurring during adolescence and as emerging adults (Gouveia et al., 2015). As adolescents internalize socialization and learning experiences, they build upon the social skills they are learning, understand the social roles they play, incorporate expectations they receive from others, and evaluate the overall abilities they develop (Schwartz, 2012). De Agrela Gonçalves Jardim et al. (2017) further found that value changes during adolescence follow predictable patterns related to an individual’s value system which further influence value-driven behaviors.

### Values and athletic participation

Chen et al. (2010) found that participation in athletics can improve health, promote prosocial values, cultivate life skills, provide social interaction, as well as enhance confidence, motivation, a sense of empowerment, and self-esteem. Ryckman and Houston (2003) found that for most individuals, school is one of the first social environments where an individual can express and apply their values. Kohn et al. (1985) found that the educational experiences of athletes and non-athletes promote intellectual openness, flexibility, and formation of self-directed values, and these experiences frequently challenge norms, expectations, and conformity to traditional values. Research has further suggested that the promotion of moral values in sport can help enhance fair play (Ring et al., 2023).

One of the primary areas of sport psychology theory and research surrounds the role of motivation in athletic performance (Gardner and Moore, 2004). Accordingly, two types of motivation that influence athletic values and performance have been articulated and examined: intrinsic and extrinsic motivation (Deci, 1975; Morris et al., 2022). Extrinsic motivation can be described as affecting behavior through external factors that are independent of personality (i.e., verbal praise, rewards, acknowledgment), whereas intrinsic motivation supports the satisfaction of needs independent of external factors from the environment, e.g., someone engaging in an activity for the pleasure and satisfaction experienced through learning, exploring, or trying something new (Vallerand, 1997).

In addition to the type of motivation employed, the type of task and motivational effort required may depend on values that the athlete perceives as most important. Task involvement represents a focus on mastering a new task in which success is judged by completing the task at hand, whereas ego involvement represents a focus on demonstrating that acquired ability in which success is determined by establishing power over others (Lee et al., 2008). However, both task and ego involvement are highly influenced by individualized values held by the athlete. For example, if a football player adopts a more task involvement focus and perceives mastering a new play as intrinsically motivating, he will be more motivated to master this task. On the other hand, if a football player adopts a more ego involvement focus, he will be more likely to master the task for external validation and praise. Overall, research has suggested that task involvement focus is considered more favorable over ego involvement focus as the former yields greater mastery skills, promotes prosocial attitudes, and mediates the effect of competence values (Lee et al., 2008).

When considering applied sport psychological practice more directly, value-driven behavior is an integral aspect of the Mindfulness, Acceptance, and Commitment (MAC) treatment framework. Briefly, this treatment modality aims to integrate mindfulness exercises, acceptance techniques, and psychological skills training programs with the aim of enhancing athletic performance through non-judgmental, present moment awareness (Gardner and Moore, 2004). This approach consists of seven modules, largely focusing on mindfulness, value identification, and behavioral implementation (Gardner and Moore, 2004). Recent meta-analytic research has shown that mindfulness-based therapeutic treatment yields large effect sizes in improving the level of mindfulness, reducing psychological anxiety, and benefitting athletic performance (Si et al., 2024). Specific to MAC, this form of treatment has been shown to increase task-focused attention (Gardner and Moore, 2007), enhance athletic performance based upon coach ratings (Moore, 2009), yield greater acceptance of negative thoughts and emotions within athletes (Dehghani et al., 2018), and show promise in both injury reduction (Zadeh et al., 2019) and enhanced athletic performance (Zadeh et al., 2019; Zhang et al., 2016). The MAC approach has also shown increased effectiveness in athletic training performance and sport-related mindfulness relative to treatment focusing solely on psychological skills training (Josefsson et al., 2019).

Within the third MAC module, athletes identify personal values and learn of the importance of acting in a manner that is congruent with these values rather than allowing their behavior to be directed by thoughts and emotions. Significant time is spent exploring the distinction between athletic outcomes or goals and values which represent the process of achieving these outcomes. The overarching purpose of the values component is to increase action in service of personal values in the athlete. These identified values are used to foster value-driven behavior and commitment to sport is necessary for goal attainment and sport enhancement (Gardner and Moore, 2004; Gardner and Moore, 2007). True adherence to value-driven behavior allows the athlete to continue training and achieve successful performances in the face of inevitable setbacks or adversity. More recent extensions to this module have suggested an alternate approach to value identification focusing on a preferred sequence of delineating team values followed by individual values (Josefsson et al., 2020) with the hope of lessening factors which may limit therapeutic engagement.

### Current study

Previous research has shown how values are shaped for individuals and develop over time. Indeed, it seems logical to think that each individual has an ordered system of personalized and prioritized values. Previous models have suggested values vary in importance, and values common across all societies include the interests and welfare of others (universalism, benevolence) versus the pursuit of one’s own interests and dominance over others (power, achievement), as well as values that emphasize independence of thought, action, and feelings (self-direction, stimulation) versus those that emphasize order, preservation of the past, and resistance to change (security, conformity, tradition) (Schwartz, 2012). However, research on the values that are relevant to athletes and the hierarchy of importance of specific athletic values is scarce. This is surprising given that Lundgren et al. (2012) found that mindfulness and acceptance-based methods such as MAC are growing in popularity and understanding and targeting positive value-driven behavior is a vital component of these interventions for sport performance enhancement. As such, the current study sought to identify commonly utilized athletic values for performance enhancement in collegiate student-athletes and to examine their perceived importance relative to one another, thus categorizing the most salient and common values utilized by them. We also sought to examine differences in perceived value importance across gender, ethnicity, sport classification, and academic standing.

## Methods

### Participants

Participants included NCAA Division I student-athletes enrolled at a private Midwestern university located within the United States. Inclusion criteria for the current study required student-athletes to be officially listed on the eligibility roster of a university sanctioned NCAA sponsored sport. A total of 180 student-athletes initiated survey protocols. From this starting point, 18 were ultimately excluded due to minimal completion of basic demographic questions. The remaining 162 participants who completed survey protocols were included within the current study.

### Procedure

Following institutional review board (IRB) and athletic department approval, surveys were distributed using the online survey tool Qualtrics and sent to student-athlete campus emails. Student-athletes were asked to complete two tasks using a previously derived list of athletic values. Utilized values were previously identified by Merz et al. (2017) after surveying student-athletes currently receiving sport psychological services regarding the personal values they perceived as of notable importance to athletic success. More specifically, these values were collected during the value-based section (module three) of the Mindfulness, Acceptance, and Commitment (MAC) (Gardner and Moore, 2004; Gardner and Moore, 2007) protocol as highlighted above. Responses were then compiled, leading to the selection of 30 unique values based upon frequency of reporting.

The first task (henceforth known as the sorting paradigm) requested that student-athletes sort all 30 athletic values (21 intrinsic and 9 extrinsic) into three separate groups with 10 values evenly placed in each group (i.e., of least importance, of average importance, of most importance) based on their perception of importance to performance enhancement and personal athletic success. Second, student-athletes were asked to rate the importance of each value independently on a 5-point Likert scale (1 = extremely important to 5 = not at all important) also based upon utilization and importance for athletic success. To prevent priming effects, the presentation of these survey tasks was counterbalanced, so that 50% of participants completed the sorting paradigm first, while the remaining 50% completed the Likert scale paradigm first. Following the completion of both survey paradigms, student-athletes answered brief demographic questions assessing age, gender, ethnicity, sport participation, and academic standing. Due to overall sample sizes, the senior and graduate student categories were combined following study completion.

### Statistical analyses

Data analyses were conducted using the Statistical Package for Social Sciences (SPSS) version 29. Participants who did not complete the survey or were missing data greater than 50% were excluded from data analysis. Prior to data analysis, data were examined for outliers and abnormal distribution characteristics. Frequency and descriptive statistics were conducted to examine age, gender, ethnicity, sport type, and academic standing in school. Given the emphasis on ordinal level data, nonparametric statistics were emphasized throughout analysis procedures. Specifically, within the sorting paradigm (i.e., ranking empirically derived values based upon sorting responses), a Friedman’s test was performed. Within the Likert scale paradigm, non-parametric Mann Whitney U analyses were conducted to examine group-level differences across obtained ordinal level data. In instances in which variables were comprised of more than two groups (e.g., academic standing), *post hoc* Kruskal-Wallis tests were performed to further examine group differences.

## Results

### Sample characteristics

A total of 162 NCAA Division I student-athletes enrolled at a private Midwestern university located in the United States participated in the current study. Student-athletes were between the ages of 18 and 30 years old (*M* = 20.12 ± 1.93), largely female (*N* = 118, 73.3%), and represented all academic grade classifications (Freshmen *N* = 42; Sophomore *N* = 44; Junior *N* = 37; Senior/Graduate Student *N* = 39). A majority of the student-athletes identified as White/Caucasian (*N* = 129; 79.6%) and the remainder Non-White/Caucasian (*N* = 33; 20.3%), including those identifying as Black/African American (*N* = 13), Hispanic or Latinx (*N* = 4), Asian or Asian American (*N* = 3), and multiracial (*N* = 13). Student-athletes participated in a variety of collegiate sports including baseball (*N* = 7), basketball (*N* = 8), cheer/dance (*N* = 10), cross country (*N* = 22), field hockey (*N* = 16), soccer (*N* = 14), softball (*N* = 12), swimming and diving (*N* = 25), tennis (*N* = 3), track and field (*N* = 39), and volleyball (*N* = 6). In terms of sport classification, 45.1% (*N* = 73) of participants were involved in team sports while 54.9% (*N* = 89) were involved in individual sports.

### Ranking of values within sorting paradigm

Due to some participants prematurely discontinuing the study and the counterbalanced nature of task presentation, incomplete surveys were encountered and total participant numbers for subsequent analyses are stated in relevant sections. To effectively rank perceived importance of empirically derived values based upon sorting responses, a non-parametric Friedman’s test was performed (*N* = 119). Notably, given the presence of 30 values, concerns regarding multiple comparisons and a significantly inflated type I error rate were anticipated. A Bonferroni correction was applied (0.05/465), rendering our significant alpha threshold at 0.0001. Table 1 provides a list of derived athletic values, as well as ranking values based upon the Friedman’s test. Note that lower scores represent a higher tendency to rate “of most importance” and higher scores represent a higher tendency to rate “of least importance.” Based upon Friedman’s test results, values were then grouped into four distinct categories. Importantly, the values within each category were not significantly different from one another; however, the values within each category were significantly different from values within other categories (*p* < 0.0001).

**Table 1:** Ranked order of athletic values.

	Athletic value	Mean rank
Tier 1	Hardworking	7.63
	Self-improvement/personal growth	8.41
	Mental toughness	8.99
	Commitment	8.90
	Having fun/enjoying the experience	10.37
Tier 2	Confidence	11.18
	Ability to learn from mistakes	11.83
	Leadership	12.09
	Positive attitude	12.19
	Personal health	12.31
	Reliability/consistency	12.52
	Trust in oneself	13.03
	Trust in others (teammates, coaches, support staff)	13.52
	Coachability/receptive to feedback	13.64
	Personal ambition	13.91
	Communication	14.50
	Physical toughness	14.56
Tier 3	Team improvement	15.48
	Selflessness/humility	15.80
	Enthusiasm	18.16
	Make family proud	18.79
Tier 4	Financial gain (scholarship money)	19.96
	Faith	20.40
	Opportunity for future success	20.45
	Make teammates happy	20.47
	Make coaches happy	21.73
	Bravery	22.32
	Travel opportunities	23.23
	Enhanced self-image/social status	23.95
	Future earnings potential	23.95

Tier 1 = “of most importance;” Tier 2 = “of average importance;” Tier 3 = “of least importance;” Tier 4 = “of no importance.”

### Ranking of values within Likert paradigm

To allow for additional in-depth analysis, student-athletes also ranked individual athletic values based on perceived importance via a 5-point Likert scale. To remain consistent with values from other analyses (i.e., Freidman’s test), lower scores represented greatest perceived importance.

Attempts were made to assess group differences across several hypotheses. Given concerns for multiple comparisons, a Bonferroni correction was again utilized, rendering our significant alpha threshold at 0.002 (i.e., 0.05/30) for subsequent analyses (Table 2).

**Table 2:** Individual rankings within Likert paradigm.

Athletic value	Mean ± SD
Hardworking	1.20 ± 0.40
Self-improvement/personal growth	1.27 ± 0.49
Mental toughness	1.36 ± 0.61
Commitment	1.43 ± 0.61
Having fun/enjoying the experience	1.46 ± 0.65
Confidence	1.53 ± 0.66
Ability to learn from mistakes	1.61 ± 0.68
Leadership	1.62 ± 0.64
Positive attitude	1.65 ± 0.65
Personal health	1.66 ± 0.70
Reliability/consistency	1.66 ± 0.68
Trust in oneself	1.73 ± 0.73
Trust in others (teammates, coaches, support staff)	1.76 ± 0.73
Coachability/receptive to feedback	1.78 ± 0.72
Personal ambition	1.81 ± 0.79
Communication	1.90 ± 0.84
Physical toughness	1.86 ± 0.75
Team improvement	1.95 ± 0.76
Selflessness/humility	2.01 ± 0.73
Enthusiasm	2.23 ± 0.65
Make family proud	2.32 ± 0.72
Financial gain (scholarship money)	2.44 ± 0.73
Faith	2.44 ± 0.75
Opportunity for future success	2.47 ± 0.73
Make teammates happy	2.47 ± 0.66
Make coaches happy	2.58 ± 0.58
Bravery	2.66 ± 0.58
Travel opportunities	2.76 ± 0.50
Enhanced self-image/social status	2.83 ± 0.43
Future earnings potential	2.84 ± 0.45

M, Mean; SD, Standard Deviation.

#### Gender

Using responses across Likert scale questions, non-parametric Mann–Whitney U tests were conducted to examine differences between genders related to the importance of individual athletic values (*N* = 151). Overall, no statistically significant differences emerged after conservative error corrections were utilized. Some non-significant trend level differences were seen between males and females across *Opportunity for Future Success* (*U* = 1,659, *Z* = 2.293, *p* = 0.022, *r* = 0.19) and *Future Earning Potential* (*U* = 1712.5, *Z* = 2.075, *p* = 0.038, *r* = 0.17), where male student-athletes placed greater emphasis than their female counterparts.

#### Ethnicity

Using responses across Likert scale questions, non-parametric Mann–Whitney U tests were conducted to examine the potential differences in the perceived importance of certain athletic values between ethnicity (i.e., White/Caucasian or Non-White/Non-Caucasian). Overall, no statistically significant differences emerged.

#### Sport classification

Using responses across Likert scale questions, non-parametric Mann–Whitney U tests were conducted to examine the potential differences in the perceived importance of certain athletic values between student-athletes engaged in team sports (e.g., baseball, basketball, soccer, volleyball, softball, field hockey) relative to those engaged in individual sports (e.g., tennis, cross country, track, swimming and diving; *N* = 153). One statistically significant difference did emerge in that student-athletes involved in team sports emphasized *Confidence* at a higher degree (*U* = 2037.50, *Z* = 3.464, *p* < 0.001, *r* = 0.28) when using conservative error corrections. Additionally, several non-significant trend level differences were seen across *Trust in Others* (*U* = 2173.50, *Z* = 2.856, *p* = 0.004, *r* = 0.23), *Team Improvement* (*U* = 2,242, *Z* = 2.568, *p* = 0.010, *r* = 0.21), *Selflessness/Humility* (*U* = 2,247, *Z* = 2.553, *p* = 0.011, *r* = 0.21), *Financial Gain* (*U* = 2,268, *Z* = 2.399, *p* = 0.016, *r* = 0.19), *Leadership* (*U* = 2305.50, *Z* = 2.351, *p* = 0.019, *r* = 0.19), *Future Earnings Potential* (*U* = 2,297, *Z* = 2.302, *p* = 0.021, *r* = 0.19), *Personal Health* (*U* = 2,332, *Z* = 2.268, *p* = 0.023, *r* = 0.18), and *Make Family Proud* (*U* = 2332.50, *Z* = 2.172, *p* = 0.030, *r* = 0.18). All trend level values were emphasized more by student-athletes involved in team sports.

#### Academic standing

*Post hoc* Kruskal-Wallis analyses were conducted to investigate whether there were significant differences in value sorting across student-athlete academic standing. Overall, no statistically significant differences emerged.

## Discussion

The current study investigated commonly utilized athletic values for performance enhancement and examined their perceived importance relative to one another, thus categorizing the most salient and common values utilized for collegiate student-athletes. We also sought to examine differences in perceived value importance across gender, ethnicity, sport classification, and academic standing. Results yielded several noteworthy findings.

This study is believed to represent the first attempt at cataloging a taxonomy of athletic values commonly utilized by collegiate student-athletes to aid with performance enhancement. Results of the value sorting paradigm suggested a hierarchy of athletic values, ranging from most important to least important, as well as a 4th category of athletic values largely perceived as unrelated to athletic performance. Intrinsically motivated values were heavily featured among the “of most importance” and “of average importance” categories, while the 8/9 extrinsic values were found in the lowest derived tiers. More specifically, among tier 3 and 4 values (see Table 1), nearly 73% (8/11) of ranked values would be categorized as extrinsically motivating. *Team Improvement* represented the only extrinsic value to be deemed “of average importance.” Overall, this suggests that student-athletes within the current sample placed significantly greater value on intrinsically motivating values and view them as far more important in achieving athletic success than extrinsically motivating values.

It is not surprising to see more intrinsically motivating athletic values ranked higher than their extrinsic counterparts. This does align with previous research in a large international cohort of male youth (mean age of 14.64 years) soccer players which suggested that task orientation was more directly related to instrument-based personal values (e.g., honesty, responsibility, integrity) while ego orientation was related to more extrinsic values (e.g., social recognition) (Berengüí et al., 2022). Student-athletes may regard intrinsic motivators as more important due to their accessibility. For example, a student-athlete may value working hard more highly than the opportunity to travel since they have far more control and individual influence over the former relative to the latter. Additionally, working hard not only increases their chances of enhancing their performance, but could also have secondary benefits if their performance improves or reflects well upon the team as whole. Second, extrinsically motivated behaviors are generally externally regulated and involve tangible rewards or accolades. Previous research has shown that while these externally regulated rewards may be perceived as motivating, the placing of too much pressure can diminish overall motivation. Specifically, Deci and Ryan (1985) found that added pressure in the form of threats, pressured evaluations, and goals imposed by another diminish intrinsic motivation by promoting an external locus of causality. Thus, student-athletes in the present study may have perceived extrinsic motivators as controlling and/or diminishing their overall autonomy.

Regarding intra-athletic differences, statistically significant differences were not seen across gender, ethnicity, or academic standing after controlling for multiple comparisons. Only a single value (*Confidence*) exhibited a statistically significant difference across team versus individual sport classification. This is not a novel finding in that previous research (Jakobsen, 2014) has also suggested a general lack of differences in intrinsic/extrinsic motivators among individual versus team sport classification. A few interesting trend level results did emerge across the current study. For example, current data suggested that male student-athletes may value *Opportunity for Future Success* and *Future Earnings Potential* more highly than their female counterparts. Results also suggested that student-athletes involved in team sports may place a higher level of importance across *Trust in Others*, *Team Improvement*, *Selflessness/Humility*, and *Leadership*. These certainly make conceptual sense. Regarding the former, male athletes have historically had more opportunities to play at the professional level than female athletes. Additionally, given the significant wage disparity between male and female athletes across all professional sports, male student-athletes are likely very aware of future opportunities they could experience being a collegiate athlete. Regarding the latter, these values all involve a team atmosphere and how an athlete might interact with other members of said team. This could support previous research suggesting that team-based sports may foster a greater sense of belonging (Bang et al., 2024) and diminished psychiatric distress (Bang et al., 2024; Pharr et al., 2019). It is important to highlight that effect sizes for these findings ranged from 0.17 to 0.23, placing them in between common thresholds to designate small (0.10) and moderate (0.30) magnitudes (Cohen, 1992), suggesting diminished clinical implications at the present moment. While we feel that these trends enhance the current study given their conceptual alignment, it is imperative to understand that while they may represent potential areas of interest for future research with larger and more diverse samples, they do not represent statistically significant results.

### Implications for clinical practice

When considering specific sport psychology interventions that emphasize value identification and foster value-driven behavior (e.g., the MAC approach), current results provide some proof of concept for a working taxonomy of potential athletic values to discuss with student-athletes who are seeking services. Gardner and Moore (2004, 2007) described the goals of the value identification exercise within the third treatment module of the MAC approach as being able to discuss how values and value-driven behavior influence one’s performance, while also achieving control of internal experiences to prepare oneself to perform at the optimum level. However, identifying personal values can be somewhat of an ambiguous process for some individuals, requiring more abstract thought relative to what they are used to. In these cases, current results could be used as a springboard to discuss common values provided by athletes to discuss if the client feels that they are relevant to their experience. Overall, results allow for the potential of a more streamlined process for both athletes and sport psychology consultants in identifying athletic-related values and the tailoring of interventions to improve athletic performance.

Second, the strong perceived relationship between intrinsically motivated values and subsequent athletic performance is encouraging as identifying and fostering intrinsic values is an integral part of sport psychological practice and serves to enhance value-driven behavior. Current findings should be viewed as mechanisms for enhancing the effectiveness of sport psychological interventions as they aim to enhance athletic performance by strengthening internal resources to aid in the maintenance of attention/focus, increase confidence, and develop coping strategies for dealing with adversity or setbacks. In essence, it directly addresses individualized intrinsic values and belief systems, strengthening them so that athletes can perform at their highest level. The results of the current study suggest that student-athletes place a high level of importance toward intrinsic values, especially regarding what they feel is helpful toward promoting athletic success. As such, the student-athletes in the current sample are indirectly validating the main goal and purpose of sport psychological interventions.

Thirdly, current results also provide guidance to the sport consultant working with an athlete who presents with a high level of extrinsically motivated values or one who appears hyper-focused on their athletic identity and the results of their performance. For example, Good et al. (1993) suggested that individuals who possess strong athletic identities place a significant amount of value in how they are perceived solely on their athletic performance. Thus, if an athlete does not perform well or is unable to compete, they may begin to struggle with their social and athletic identities. Furthermore, athletes who suffer a serious or chronic injury and must undergo physical rehabilitation often experience elevated levels of depression and mood dysfunction (Brewer et al., 2010). By tailoring interventions to focus on specific mental skills training, coping strategies, and mindfulness work, student-athletes may see more of an improvement in their performance rather than focusing on one’s social status, playing time, individual stats, etc. However, the first step of this process would likely include working with the athlete to transition their focus to more intrinsically motivating values. The current results are believed to assist consultants in this process by providing credibility to the consultant’s claims emphasizing the importance of controlling what can be controlled (i.e., intrinsic motivators) and opening up new avenues of internal, thought-based growth and performance enhancement. By doing so, this would ultimately provide student-athletes the tools and skills to deal with challenges that may arise, while also utilizing these skills beyond athletics.

Finally, largely null findings across ethnicity comparisons could suggest that both white/Caucasian and non-white/Caucasian student-athletes utilize similar values and may approach athletic performance and performance enhancement in similar rather than dissimilar ways. This could also be said about class standing. Subsequently, this knowledge would allow clinicians to cast a broader net when assisting athletes in identifying important personal values. However, given the sparse research on these topics, further inquiries are necessary.

### Limitations and future considerations

The current study has several limitations which should be considered. First and foremost, while our overall sample size may be appropriate for an initial exploratory study, it is likely that some analyses were under-powered. This may also explain the resultant trend-level findings across several group-based comparisons. While these trends largely made conceptual sense and are believed to somewhat validate our findings, it is imperative that the current study be replicated with a larger sample size to see if current results hold. Second, the current study was under-represented across several major sports, including baseball, basketball, football, and soccer/European football. Future research should focus on data acquisition from larger university settings and strive for more diversity across sport types. This is especially important as the sports mentioned above all have higher earnings potential and opportunities for professional level participation, meaning that extrinsic values could be more influential in this sample. However, this is speculative at this point and needs to be further examined. Third, there remains the possibility that student athletes identified values which they deemed more socially acceptable rather than their true beliefs. Future research may wish to include a social desirability scale to assess the degree this sort of behavior may have occurred. Finally, female student-athletes comprised 73.3% of the current sample, while White/Caucasian student-athletes comprised 79.6% of the current sample. While this represents the demographics of the university which this data was obtained reasonably well, there are generalizability concerns surrounding the larger body of NCAA student-athletes, as well similarly-aged athletes across the world. The current study also did not assess socioeconomic status (SES) among student athletes or nationality/cultural upbringing. Future research should focus on replication and expansion in a more demographically and culturally diverse sample.

## Conclusion

The current study sought to examine and delineate a taxonomy of values based on their perception of importance relative to athletic success in a group of collegiate student-athletes. Overall, the results yielded a useful summary of ranked list of athletic values, with a strong preference toward intrinsically motivating values rather than extrinsically motivating values. These findings have several noteworthy clinical implications, including the validation of sport psychological practices. They also benefit clinicians working with student-athletes by providing a springboard to discuss values important in their lives and how to transition personal mindsets toward value-driven behavior with the goal of eventual sustained athletic success.

## Data Availability

The raw data supporting the conclusions of this article can be made available by the authors upon reasonable request.
